# Supplementary material for: Resuscitation and Forensic Factors Influencing Outcome in Penetrating Cardiac Injury
Source: Diagnostics (Basel). 2024 Jul 1;14(13):1406. doi: 10.3390/diagnostics14131406 (PMC11241016; doi:10.3390/diagnostics14131406)
Supplement: Supplementary file 1 [file diagnostics-14-01406-s001.zip › diagnostics-3069634-supplementary.pdf]

SUPPLEMENTARY MATERIAL for

Resuscitation and Forensic Factors Influencing Outcome in Penetrating Cardiac Injury.

TABLE OF CONTENTS

Supplementary S1 - National social security (CCAM) codes used for extraction of patient data and the corresponding ICD-10 codes. *3*

Supplementary S2 – Table of data *5*

Supplementary S3 - Characteristics of autopsied patients *9*

Supplementary S4 - Scale details *10*

Supplementary S1 - National social security (CCAM) codes used for extraction of patient data and the corresponding ICD-10 codes.

**CCAM codes**	**ICD-10 code**
S218	Open wound of other parts of thorax
S250	Injury of thoracic aorta
S251	Injury of innominate or subclavian artery
S252	Injury of superior vena cava
S254	Injury of pulmonary blood vessels
S255	Injury of intercostal blood vessels
S257	Injury of multiple blood vessels of thorax
S2580	Injury of azygos vein
S2588	Injury of other blood vessels of thorax except azygos vein
S259	Injury of unspecified blood vessel of thorax
S260	Injury of heart with hemopericardium
S2600	Injury of heart with hemopericardium without open wound into thoracic cavity
S2601	Injury of heart with hemopericardium with open wound into thoracic cavity
S268	Other injuries of heart
S2680	Other injuries of heart without open wound into thoracic cavity
S2681	Other injuries of heart with open wound into thoracic cavity
S269	Injury of heart, unspecified
S2690	Injury of heart, unspecified, without open wound into heart
S2691	Injury of heart, unspecified, with open wound into heart

S271	Traumatic haemothorax
S311	Open wound of abdominal wall
S390	Injury of muscles and tendons of the abdomen, lumbar area, pelvis
T145	Injury of blood vessel(s) of unspecified body region
W260	Contact with knife, sword or dagger
X780	Intentional self-harm by sharp object, home
X789	Intentional self-harm by sharp object, unspecified place
X849	Intentional self-harm by unspecified means, unspecified place
X99	Assault by sharp object
X990	Assault by sharp object, home
X991	Assault by sharp object, residential institution
X992	Assault by sharp object, school, other institution and public administrative area
X994	Assault by sharp object, street and highway
X995	Assault by sharp object, trade and service area
X998	Assault by sharp object, other specified places
X999	Assault by sharp object, unspecified place

Supplementary S2 – Table of data

**Type of data**	Data	Patients evaluated
**Prehospital**	Surviving or deceased	Survivors and autopsied patients
	Sex	
	Age at emergency management or at death	
	Intervention of emergency services and time to arrival	
	Resuscitation attempted or not	
	Clinical signs (HR, BP, hypotension, Glasgow score, limb movement, spontaneous ventilation)	
	Transfusion of FFP and packed RBC, chest tube placement	
	Intubation, catecholamines required	
	Volume expansion > 1 L	
	Cardiorespiratory arrest	
	External heart massage and duration	
	Prehospital death	
	Response time of emergency services and transport to hospital	

**In-hospital**	Clinical signs on arrival (HR, BP, hypotension, Glasgow score, state of shock)	Survivors and autopsied patients
	Intubation and mechanical ventilation	
	Catecholamines required	
	Volume expansion > 1 L	
	Chest tube placement	
	Packed RBC and FFP transfusion	
	Haemoglobin level on arrival	
	Pre- or peroperative cardiorespiratory arrest	
	External cardiac massage and duration	
	Paraclinical investigations (laboratory tests, cardiac ultrasound, pre- and postoperative CT)	
	Pericardial effusion, tamponade, pneumothorax or haemothorax	
**Operative**	Type and duration of surgery	Survivors and autopsied patients
	Presence of peroperative adrenaline	
	Blood loss	
	Catecholamines required	
	Packed RBC or FFP transfusion	
	Description and size of injuries	
	Death	

**Postoperative**	Sedation and adrenergic support	Survivors and autopsied patients
	Duration of stay in resuscitation or intensive care and in hospital	
	Wounding agent recovered	
	Death	
Injury characteristics	Mechanism of injury	Survivors and autopsied patients
	Self-inflicted or other-inflicted injury	
	Location, number and size of injuries	
	Associated injuries	
Post-mortem	At-the-scene examination of the body, physical examination or autopsy	Autopsied patients
	Location, number and size of injuries at autopsy	
	Complementary investigations (CT, pathology, toxicology)	
	Pre-existing cardiac condition	
	Pathologist estimation of survival time	
	Cause of death	
	Wounding agent recovered	

Scores	Simplified Severity Index II (Index de Gravité Simplifié II, IGS2 (probability of death in hospital)	Survivors and autopsied patients evaluated
	Injury Severity Score, ISS	
	Revised Trauma Score, RTS	
	Sequential Organ Failure Assessment, SOFA (probability of death)	
	American Association for the Surgery of Trauma-Organ Injury Scale (AAST-OIS) (cardiac injury severity score)	

HR : heart rate, *BP* : blood pressure

Supplementary S3 - Characteristics of autopsied patients

**Variables**	N=30
On-scene examination	14
Physical examination	1
Number of injury systems, n	2 (2-6)
Anterior thoracic injury systems, n	1 (1-2)
Cardiac injury systems, n	1 (1-1)
Fatal injury systems, n	1 (1-1)
**Location of fatal injury systems**	
Heart	24 (80.0)
Heart and large vessels	3 (10.0)
Large vessels	2 (6.7)
Heart and lung	1 (3.3)
**Cause of death (autopsy, pathology, study of medical records if present)**	
Haemorrhagic shock	23 (76.7)
Multiorgan failure	4 (13.3)
Tamponade	3 (10.0)
**Pathology report**	
Report made	18 (60)
Estimation of survival time	9 (47.4)

Post-mortem toxicology	
Toxicology tests carried out	19 (63.3)
Alcohol intoxication	12 (48.0)
Cannabis intoxication	7 (28.0)
Other intoxication	5 (20.0)

Categorical data are expressed as n(%) and numerical data as medians (25th-75th percentiles)

Supplementary S4 - Scale details

In our study, various grading and prognostic scores were taken into account (from medical records or calculated a posteriori with medical record data): ISS and RTS and an anatomical severity score was used for cardiac lesions: the Organ Injury Scaling of the American Association for the the Surgery of Trauma (OIAS-AAST).

Injury Severity Score: ISS

The Injury Severity Score or ISS is a medical score used to assess the severity of a polytrauma patient. It is correlated with mortality, morbidity and hospitalization time after trauma.

It is calculated by estimating the severity of injuries (based on the AIS score) in

6 regions of the body. Injury severity is rated from 0 (minor) to 6 (maximal).

The score ranges from 0 to 75. If one of the lesions is rated at 6, the score is automatically set at 75. Trauma is considered major if the score is > 15

anatomical region	severity of injury
Head and neck	1 Minor
Face	2 Moderate
Chest	3 Serious
Abdomen	4 Severe
Extremity (Including Pelvis)	5 Critical
External	6 Unsurvivable

References

Baker SP, O'Neill B, Haddon W Jr, Long WB. The injury severity score: a method for describing patients with multiple injuries and evaluating emergency care. Journal of Trauma 1974, 14 (3): 187-96

Revised Trauma Score

The RTS has been designed and tested for pre-hospital assessment of polytrauma patients.

The RTS is one of the more common scores aimed to measure the functional consequences of an injury. It uses three specific physiologic parameters: (1) the

Glasgow Coma Scale (GCS); (2) systemic blood pressure; and (3) the respiratory rate. It also provides an estimate of the patient's probability of survival. The score ranges from 0 to 8, with a survival probability of 2.7 for a score of a score of 0 and 98.8% for a score of 8

Reference:

Champion HR, Sacco WJ, Copes WS, Gann DS, Gennarelli TA, Flanagan ME. A revision of the Trauma Score. J Trauma. mai 1989;29(5):623-9.).

Organ Injury Scaling of the American Association for the Surgery of Trauma (OIAS-AAST)

Organ Injury Scaling of the American Association for the Surgery of Trauma (OIASAAST) is an anatomical score used to assess the severity of penetrating trauma.

Grade	Injury description
I	Penetrating pericardial wound without cardiac injury, cardiac tamponade, cardiac herniation
II	Penetrating tangential myocardial wound up to, but not extending through, the endocardium, without tamponade
III	Penetrating tangential myocardial wound up to, but not extending through the endocardium, with tamponade
IV	Penetrating cardiac injury with septal rupture, pulmonary or triscuspid valvular incompetence, papaillary muscle dysfunction or distal coronary artery occlusion causing cardiac failure.
	Penetrating cardiac injury with aortic or mitral valve incompetence
	Penetrating cardiac injury of the right ventricle, right atrium, or left atrium
V	Penetrating cardiac injury with proximal artery occlusion
	Penetrating left ventricular perforation
	Stellate wound with < 50% tissue loss of the right ventricle, right atrium, or left atrium
VI	Penetrating wound producing > 50% tissue loss of a chamber

Reference :

Moore EE, Malangoni MA, Cogbill TH, Shackford SR, Champion HR, Jurkovich GJ, et al. Organ injury scaling. IV: Thoracic vascular, lung, cardiac, and diaphragm. J Trauma. mars 1994;36(3):299-300.) is an anatomical score used to assess the severity of penetrating cardiac trauma.
